# Breads Fortified with Freeze-Dried Vegetables: Quality and Nutritional Attributes. Part II: Breads Not Containing Oil as an Ingredient

**DOI:** 10.3390/foods5030062

**Published:** 2016-09-08

**Authors:** Viren Ranawana, Fiona Campbell, Charles Bestwick, Phyllis Nicol, Lesley Milne, Garry Duthie, Vassilios Raikos

**Affiliations:** Rowett Institute of Nutrition and Health, University of Aberdeen, Aberdeen AB25 2ZD, Scotland, UK; fiona.campbell@abdn.ac.uk (F.C.); c.bestwick@abdn.ac.uk (C.B.); p.nicol@abdn.ac.uk (P.N.); l.milne@abdn.ac.uk (L.M.); g.duthie@abdn.ac.uk (G.D.); v.raikos@abdn.ac.uk (V.R.)

**Keywords:** bread, vegetables, macronutrient oxidation, storage, digestion

## Abstract

The present article describes the second part of a study investigating the effect of adding vegetables on the nutritional, physico-chemical, and oxidative properties of wheat bread, and specifically focuses on bread that does not contain oil as an added ingredient. Wheat flour breads fortified with freeze-dried carrot, tomato, beetroot or broccoli were developed and assessed for their nutritional composition, antioxidant potential, oxidative stability, and storage properties. Using a simulated in vitro model, the study also examined the impact of vegetable addition on the oxidative stability of macronutrients during gastro-intestinal digestion. Adding vegetables improved the nutritional and functional attributes of the oil-free breads. However, they demonstrated a lower antioxidant potential compared to their oil-containing counterparts. Similarly, the textural and storage properties of the oil-free vegetable breads were poorer compared to the oil-containing breads. As expected, in the absence of oil the oil-free breads were associated with lower lipid oxidation both in their fresh form and during gastro-intestinal digestion. Adding vegetables reduced protein oxidation in the fresh oil-free breads but had no effect during gastro-intestinal digestion. The impact of vegetables on macronutrient oxidation in the oil-free breads during digestion appears to be vegetable-specific with broccoli exacerbating it and the others having no effect. Of the evaluated vegetables, beetroot showed the most promising nutritional and physico-chemical benefits when incorporated into bread that does not contain added oil.

## 1. Introduction

There is presently growing emphasis on reformulating processed foods to make them healthier. Increasing consumer demands for healthier foods has resulted in a drive towards producing ‘clean label’ products that are nutritionally superior and do not contain synthetic additives. Macronutrient oxidation is a particular concern for the food industry as it adversely affects organoleptic properties and shelf life, and next to microbes it is the second greatest contributor to food spoilage [1]. Most processed foods, therefore, have synthetic additives to control oxidation, despite increasing consumer pressure to reduce their use.

The oxidation of macronutrients in foods has implications from health, nutritional, and food science perspectives as highlighted in the previous article describing part 1 of this study [2]. The oxidation of fats and proteins lead to the development of toxic end-products that contribute to disease pathogenesis by affecting the stability and biochemistry of cells and genes. Thus, it is imperative that dietary macronutrients are protected against oxidation. Plant products are rich in natural antioxidants and functional components which have been suggested to curtail oxidation of foods [1]. In agreement, work from our group showed that adding vegetables to burgers and emulsions improved their oxidative stability (resistance to oxidation) and shelf life [3,4,5]. However, as antioxidants in vegetables are sensitive to food processing conditions, their efficacy in processed food systems needs to be evaluated product-specifically.

In this context, the present series of papers investigated the effect of adding vegetables on the oxidative stability, nutrition, and quality of wheat-flour bread. The first paper [2] investigated the effects of vegetable addition into bread that had fat as an added ingredient. Modern-day commercial breads often have added fat as it helps improve texture and sensory attributes [6]. The most common fats used are those of vegetable origin such as palm, sunflower, rapeseed, and corn. However, fat is not an essential ingredient for making bread; indeed, traditional breads such as baguettes, cottage loaves, and flat bread are typically made without it. As consumer demands for healthier breads increase [7] there is growing emphasis on developing breads that are low in energy, nutritionally rich, and free of synthetic additives. 

The first part of this study [2] showed that the addition of vegetable powders to bread containing oil as an ingredient significantly improved nutritional and antioxidant properties of bread whilst also enhancing shelf life. This article forms the second part of the study and assessed the impact of vegetable addition in breads that do not have fat as an added ingredient. The objective of this study was to assess the nutritional attributes of these vegetable breads, their oxidative stability during storage and in vitro gastrointestinal digestion, and their shelf life stability. The study hypothesised that adding vegetables will improve the nutritional attributes of bread, and their oxidative stability during storage and gastrointestinal digestion.

## 2. Materials and Methods 

### 2.1. Test Breads

Five bread types were prepared (plain (control), carrot, tomato, broccoli, and beetroot) at the Rowett Institute of Nutrition and Health (RINH). The ingredients used for the plain bread were strong white wheat flour (60% *w*/*w*; Allinson flour, Peterborough, UK), water (37%), yeast (1.8%; Tesco Stores Ltd., Dundee, UK) and salt (1.2%). The vegetable breads contained the same ingredients with the exception that 10% of the flour (by weight) was substituted with freeze dried vegetable powder. This level of substitution was based on initial developmental work which showed that 10% was the optimum replacement level that did not affect the physical properties of bread. All the breads were prepared using the same methodology as follows: the yeast was dissolved in the water (pre-warmed to 40 °C), added to the dry ingredients, and mixed to form doughs. The doughs were kneaded by hand for ten minutes and subjected to a first prove for 25 min at room temperature. They were then knocked back, made up, placed in bread tins, and proved for a further 40 min and baked at 200 °C for 30 min in a non-fan assisted oven. This time temperature combination was based on typical conditions used for bread baking [8]. The cooled breads were sliced into 1.5 cm thick slices and half of the loaf was used for texture analyses and shelf life stability. The other half was freeze dried (Model HS1, Frozen in Time Ltd., York, UK), ground to a fine powder (<400 µm) (model ZX809X, Wahl, Kent, UK), and stored in black polyethylene bags at −70 °C, and used for all other analyses. 

The freeze dried vegetable powders were prepared at RINH. Fresh carrot, tomato, broccoli, and beetroot were purchased from local supermarkets. These were washed, chopped, freeze dried (HS1, Frozen in Time Ltd., York, UK), ground to a fine powder (<250 µm) (Blixer 2, Robot Coupe, Montceau-en-Bourgogne, France), and stored in black polyethylene bags at 6 °C until used for making the breads. The broccoli was separated into florets before drying. The tomato was dried with the seeds, and the carrot and beetroot were dried without peeling.

Routine analytical procedures were followed in triplicate to determine the proximate composition of the breads [9]. In addition, several micronutrients and non-nutrients with recognised antioxidant activity (vitamin E: α and γ-Tocopherol, and carotenoids: α-Carotene, β-Carotene, β-Cryptoxanthin, Lutein, Lycopene) were quantified using high performance liquid chromatography [10].

The chemicals, materials and methods used in the present study have been described in detail elsewhere [2]. Therefore, elaboration of the methods within this article has been limited to the salient steps. 

### 2.2. Antioxidant Capacity of Vegetable Breads

Aqueous extracts of the bread samples were made by homogenising 0.4 g of freeze dried bread with 9.6 mL of 0.9% NaCl. Aqueous extracts were used as they were physiologically more relevant than ethanolic extracts. The method of Benzie and Strain [11] was used for assaying Ferric Reducing Antioxidant Potential (FRAP), and the method of Ou et al. [12] for assaying Hydroxyl Radical Averting Capacity (HORAC). The HORAC assay was carried out using a kit (Product number TA30, Oxford Biomedical Research Inc., Rochester Hills, MI, USA). NaCl at an isotonic concentration was used as it was deemed to be a more physiologically relevant extraction medium. Antioxidant capacity of each bread type was assayed in four independent replicates and the data was pooled for analysis.

### 2.3. Oxidation End-Products in Vegetable Breads

Lipid oxidation end-products in fresh bread were measured as Thiobarbituric Acid Reactive substances (TBARS) and protein carbonyls (PC). Freeze dried bread samples (~50 μg) were accurately weighed and added to 4 mL of distilled water and homogenised (Ultra-Turrax T18, Janke & Kunkel, IKA instruments, Staufen, Germany) at 12,000 rpm for 10 s. The homogenates were analysed for TBARS and PC as previously described [2] in four replicates. For TBARS analysis, 1 mL of Thiobarbituric Acid (TBA) reagent (0.67% TBA solution mixed in an equal volume of glacial acetic acid) was added into each tube and vortexed. The tubes were subsequently heated at 95 °C for 30 min, cooled to room temperature, and centrifuged for 10 min at 3500 rpm (CR 312, Jouan, Thermo Fisher Scientific, Waltham, MA, USA). The supernatants were analysed for TBARS content by reverse-phase High Performance Liquid Chromatography (HPLC) using a Waters 2695 separations module coupled with a Waters 2475 fluorescence detector (Waters Ltd., Elstree, UK) and a 100 × 4.6 Phenomenex Luna 5 μ C 18(2) 100 A column set at 30 °C (Phenomenex, Cheshire, UK). Separations were carried out on 20 L samples at a flow rate of 0.8 mL/min over 20 min using a mobile phase made of 60% 50 mM KH_2_PO_4_ (pH 7.0) and 40% Methanol. Standard solutions of 1,1,3,3 Tetramethoxypropane were simultaneously analysed in triplicate for constructing calibration curves and quantification of TBARS. TBARS content was expressed as nmol per g of sample. Protein carbonyls were measured using the following method. Following incubation (25 °C; 10 min) of the samples (0.1 g) with KCl (0.15 M; 1 mL) containing FeSO_4_ (1 mM) and H_2_O_2_ (1 mM), 20% TCA was added to precipitate the protein. Following the addition of 0.2% DNPH, the samples were heated at 45 °C for 1 h and centrifuged at 13,000 *g* for 5 min. The supernatant was removed and the pellet was washed in ethanol:ethyl acetate (1:1 *v*/*v*) three times, dissolved in 300 L 6 M guanidine hydrochloride, and absorbance read at 370 nm. Protein content was determined using the Pierce 660 protein assay (Thermo Scientific, Paisley, UK) according to the manufacturers recommended protocol. Carbonyl content was quantified using the molar extinction coefficient of 22,000/M cm and the results expressed as nmol of carbonyl per mg of protein. 

### 2.4. Changes in Texture during Storage

Freshly baked and cooled loaves were sliced into 1.5 cm slices on the day of baking, and pairs of randomly selected slices were placed in polyethylene bags. The bags were stored at room temperature (20 °C) in the dark. Texture profile analyses of the bread crumb was carried out at 0, 1, 2, and 4 days of storage using a texture analyser (CT3, Brookefield Viscometers Ltd., Harlow, UK) equipped with a cylinder probe (TA11/1000, D = 25.4 mm) as described in the first part of this paper [2]. Each bread slice was 40% compressed twice to give a two bite texture profile. Two slices were tested for each treatment at each time point which resulted in four readings per treatment per day. Trigger load and test speed were 10 g and 0.5 mm/s respectively. The parameters hardness, cohesiveness, and gumminess were evaluated using the in situ software (TexturePro CT, Brookefield Viscometers Ltd., Harlow, UK). Duplicate measures were made for each bread type on each day.

### 2.5. Oxidative Stability During in Vitro Gastrointestinal Digestion

#### In Vitro Gastrointestinal Digestions

In vitro gastro-intestinal digestions were carried out using a system that mimics the oral, gastric, and intestinal phases of human digestion. The method used is described in detail in the article constituting part one of this study [2]. Briefly, the samples were weighed into 15 mL black centrifuge tubes and 3 mL of cold simulated saliva was added. The digestion tubes were then placed in the shaking water bath for 5 min to complete the oral phase of digestion (pH 6.8) and the phase halted by the addition of 0.5 mL of 0.3 M HCl. The gastric phase of digestion was initiated by the addition of 8 mL of double-strength SGF (pH 2.0) containing 0.68 mg of Ascorbic acid, 0.11 mg of FeSO4 and 6.8 mg of ADP. Lipid peroxidation is induced by Fe/ADP complex in the presence of Ascorbic acid and was thus added to create a pro-oxidant environment. The gastric digestion phase was continued for four hours. At the end of two hours of gastric digestion, 1.5 mL volumes of gastric digesta were transferred into separate tubes containing 1.5 mL of double strength SIF (pH 8.0) and the intestinal digestion phase was carried out in parallel for two hours. At baseline, and during each of the three digestion phases, 0.5 mL digesta aliquots were aspirated and transferred into, (a) glass tubes containing 1 mL 20% Trichloroacetic acid (TCA) for measuring concentrations of Thiobarbituric acid reactive substances (TBARS) and, (b) glass tubes containing 50 µL of 0.2% Dinitrophenylhydrazine (DNPH, in 3.5 M HCl) for measuring PCs. All of the bread samples were subjected to in vitro digestions in three independent runs and the data was pooled for analysis. 

For measuring TBARS the total volume of the TCA tubes containing digesta samples was brought to 4 mL with double-distilled water before analysing for TBARS. The TBARS and PC content in the digesta samples were analysed as previously described [2]. 

### 2.6. Statistical Analyses

Total TBARS and PCs formed during four hours of in vitro gastric digestion and two hours of intestinal digestion were quantified by calculating the Areas Under the digestion Curves (AUC) using the trapezoidal rule. Data on the TBARS and PC contents in the fresh bread samples, AUCs from gastrointestinal digestions, and the antioxidant capacity and texture analysis data were analysed using one-way ANOVA. Post hoc tests were carried out where significant differences were observed using the Bonferroni correction, Ryan, Einot, Gabriel, and Welsch Q procedure, and Scheffe test as appropriate. A *p* < 0.05 was considered statistically significant.

## 3. Results

### 3.1. Nutritional Composition of Vegetable Breads

**Figure 1:**
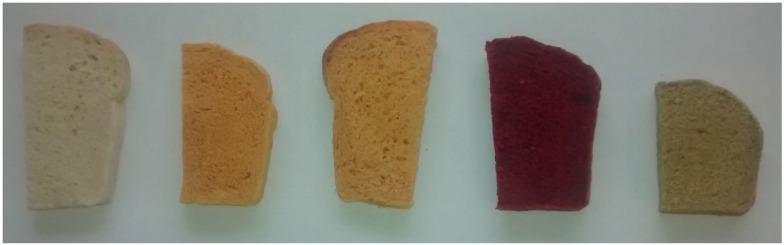
Bread samples showing transverse structure and loaf size. From Left to right: Plain bread, Carrot bread, Tomato bread, Beetroot bread, Broccoli bread.

The addition of vegetables produced statistically significant changes in the nutritional profile of breads compared to the control (Table 1). Compared to the plain bread, broccoli addition significantly increased protein content whilst the other vegetables reduced it (*p* < 0.05). Adding vegetables significantly attenuated the total carbohydrate content of bread and significantly increased non-starch polysaccharides (compared to the plain bread) (*p* < 0.05). Broccoli and tomato significantly increased fat content, however, in practical terms it was by a small amount (0.2 g/100 g). Adding vegetables also significantly increased the total mineral content of the breads compared to the plain bread. Adding broccoli and tomato significantly increased the vitamin E content of breads compared to the plain bread and this increment was substantial. As expected, the addition of vegetables significantly increased the carotenoid content of bread (*p* < 0.05). Figure 1 illustrates the physical appearance of the breads.

### 3.2. Antioxidant Capacity of Vegetable Breads 

**Figure 2:**
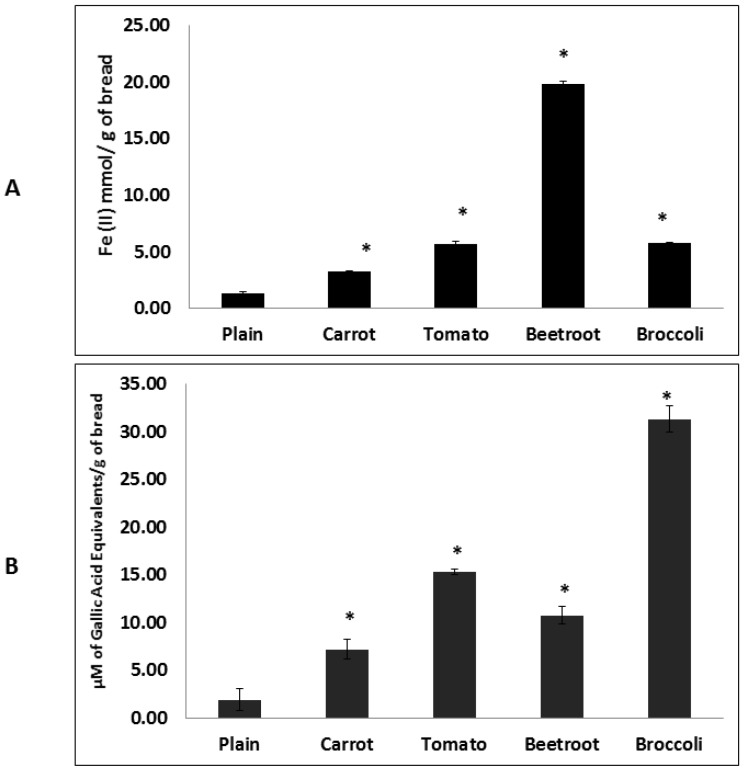
Antioxidant capacity of breads measured using FRAP (**A**) and HORAC (**B**) assays. FRAP: Ferric Reducing Antioxidant Potential; HORAC: Hydroxyl Radical Averting Capacity. Columns with asterisks are significantly different to the plain bread (*p* < 0.05; *n* = 4). Error bars are standard errors.

Adding vegetables significantly affected the FRAP assay associated antioxidant capacity of the vegetable breads (F(4,14) = 1793.59; *p* < 0.001) (Figure 2A). Post hoc analyses showed that all the vegetable breads had significantly higher antioxidant capacities compared to the plain bread. While carrot bread had the lowest antioxidant capacity of all the vegetable breads (3.2 ± 0.2 Fe(II) mmol/g of bread), broccoli and tomato breads demonstrated similar levels (5.8 ± 0.1 and 5.6 ± 0.5 Fe(II) mmol/g of bread, respectively). Beetroot bread had the greatest antioxidant potential and was significantly higher than the others (19.9 ± 0.4 Fe(II) mmol/g of bread). 

The HORAC assay also showed significant differences in the antioxidant potential of the breads (F(4,14) = 121.65; *p* < 0.001) (Figure 2B). Post-hoc analyses indicated that all the vegetable breads had significantly greater antioxidant potentials compared to the plain bread. Broccoli bread had the significantly highest antioxidant potential of all the treatments (31.3 ± 2.3 µmol GA Eq/g of bread) followed by tomato (15.3 ± 0.5 µmol GA Eq/g of bread). Carrot and beetroot breads showed similar antioxidant capacities (7.2 ± 1.8 and 10.8 ± 1.6 µmol GA Eq/g of bread, respectively). 

### 3.3. Oxidation End-Products in Vegetable Breads

**Figure 3:**
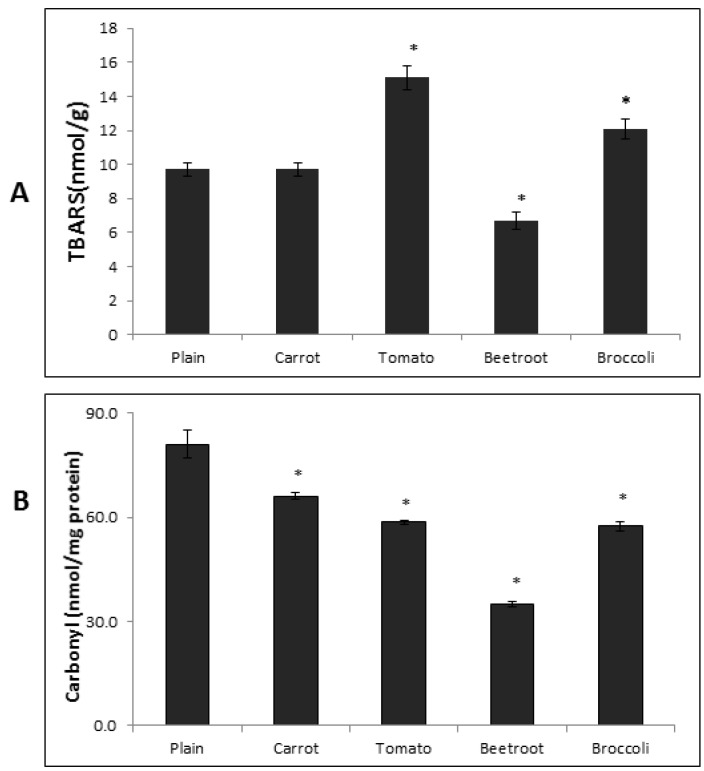
TBARS (**A**) and Protein Carbonyl (**B**) contents in the fresh breads. TBARS = Thiobarbituric acid reactive substances. Columns with asterisks are significantly different to the plain bread (*p* < 0.05; *n* = 4). Error bars are standard errors.

The TBARS content in the fresh breads showed significant differences (F(4,15) = 25.42; *p* < 0.001) (Figure 3A). Beetroot bread contained the significantly lowest TBARS content (6.7 ± 1 nmol/g) of all the breads. The plain and carrot breads had similar TBARS contents (9.74 ± 1 and 9.69 ± 1 nmol/g, respectively) but were significantly different to all the others. The tomato and broccoli bread contained the highest and second highest TBARS contents, respectively (15.13 ± 2 and 12.08 ± 1 nmol/g) and were significantly different to each other and all other breads. 

The PC content in the fresh breads also differed significantly (F(4,10) = 217.71; *p* < 0.001) (Figure 3B). The plain bread contained the highest PC content and was significantly higher than all the vegetable breads (81.1 ± 4 nmol/mg protein). The beetroot bread had the (significantly) lowest PC content (34.9 ± 1 nmol/mg protein) followed by tomato and broccoli breads which had comparable amounts (58.6 ± 1 and 57.4 ± 1 nmol/mg protein, respectively) that were significantly different to all other breads. The carrot bread contained the second highest PC content and was significantly different to all the other breads (66.1 ± 1 nmol/mg protein). Correlational analysis showed that there was no association between TBARS and PC contents in the breads (*r* = 0.30, *p* = 0.62).

### 3.4. Changes in Texture during Storage

All five breads increased in hardness during storage. The hardness of carrot and tomato breads (5.01 ± 0.8 N and 6.56 ± 0.3 N, respectively) were similar to that of the plain bread on the day of baking (4.45 ± 1.2 N) (Table 2). Broccoli and beetroot breads were significantly harder (15.72 ± 1.3 N and 8.37 ± 1.6 N, respectively) compared to the plain bread (*p* < 0.05). However, by the fourth day of storage only broccoli bread (22.00 ± 2.4 N) showed a significantly greater degree of hardness compared to the plain bread (10.9 ± 1.3 N) Cohesiveness showed a decreasing trend during storage and all breads showed a similar degree on the day of baking (*p* > 0.05). Similarly, all the breads had a comparable degree of cohesiveness at the end of four days of storage. Gumminess demonstrated an increasing trend during storage and carrot, tomato and beetroot breads showed similar values to the plain bread on the day of baking (*p* < 0.05). Broccoli bread had a significantly higher gumminess on the day of baking (6.90 ± 0.9 N) compared to all the other breads (*p* = 0.001). However, all the breads showed similar levels of gumminess after four days of storage (*p* > 0.05). Values for the plain bread were taken as the comparator. 

### 3.5. In Vitro Gastrointestinal Digestions 

**Figure 4:**
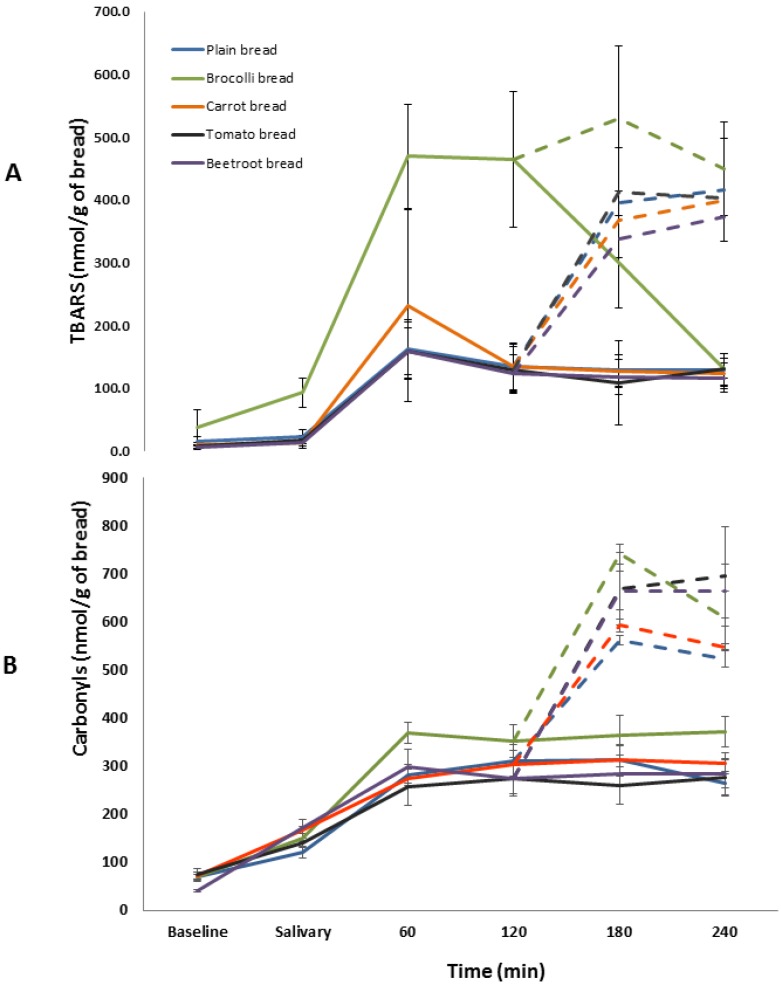
Temporal changes in TBARS (**A**) and protein carbonyls (**B**) production during gastro-intestinal digestion of the breads. Solid lines represent the salivary and gastric phases of digestion. The broken lines represent the intestinal phase of digestion. Values are means ± SE (*n* = 6).

The amount of TBARS generated during the oral phase of digestion was significant (F(4,25) = 25.23, *p* < 0.001) (Figure 4). Post hoc analyses showed that broccoli bread generated a significantly higher amount of TBARS whilst all other vegetable breads produced similar amounts to the plain bread. Significant differences in TBARS were observed during gastric digestion of the vegetables breads (F(4,25) = 31.36, *p* < 0.001). Broccoli bread produced a significantly greater amount of TBARS compared to the plain bread whilst all the other vegetable breads generated similar amounts. Significant differences were also observed in TBARS during the intestinal phase of digestion (F(4,25) = 9.83, *p* < 0.001) (Figure 4). Similar to the earlier two phases, only broccoli bread showed a significantly greater amount compared to the plain bread. Statistical analysis within each of the time points also showed that broccoli bread was associated with significantly greater amounts of TBARS (*p* < 0.01) and that all the other breads contained similar amounts (*p* > 0.05). 

**Figure 5:**
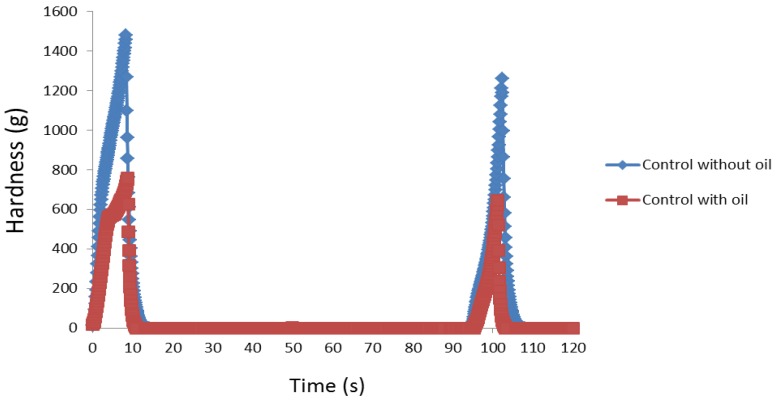
Representative graphical representation of the effect of oil on the firmness of bread crumb after four days of storage.

The PC content in all the vegetable breads was statistically similar to the plain bread (*p* > 0.05) (Figure 5). The total amount of PCs generated during the salivary, gastric, and intestinal phases were not significantly different between the breads (F(4,25) = 1.07; *p* = 0.39, F(4,25) = 1.36; *p* = 0.28, and F(4,25) = 1.70; *p* = 0.18 respectively). 

## 4. Discussion

To our knowledge no studies have attempted to systematically study the effects of vegetable addition on the oxidative stability of bread and compare effects in the absence and presence of oil. Whilst the first part of this series dealt with breads containing oil as an added ingredient [2] this paper reports data for breads that do not have added oil. We felt it prudent to study both types of bread considering the central role fat plays in the oxidative stability and quality of bread and the wide consumption of both types. We also felt that it would be scientifically compelling to compare the impact of vegetables in these two bread types from nutritional, oxidative stability, and physico-chemical aspects. The specific vegetables tested in this study were selected based on findings from previous research by our group showing them to be promising in terms of nutritional and antioxidant properties [3]. 

Even though it is widely thought that the digestive system is an anaerobic environment, there is evidence to indicate that the alimentary tract can often be oxygen rich [13]. Mastication has also been shown to oxygenate food [14], and, therefore, it is likely that ingested food is exposed to pro-oxidant conditions during digestion. Hence, it is imperative that macronutrients in the chyme are protected from oxidation so that they may be metabolised in the intended manner. The oxidation of lipids and proteins generate products such as TBARS, carbonyls, and glycation end-products that provoke morbidity [15,16], and antioxidants in food could play a significant role in protecting macronutrients from oxidation during digestion. Given the equivocal evidence often seen in clinical studies investigating the metabolic effects of absorbed dietary antioxidants [17], they may be playing a more important protective role within the digestive lumen. 

Overall, the addition of vegetables improved the functional and nutritional properties of bread, and this agrees with previous work [18,19,20]. Adding vegetables significantly increased NSP, ash, and carotenoid contents as could be expected. Contrary to what could be expected, the lycopene content in the oil-free tomato bread was lower (54.6 µg/g of dry matter) than in its oil-containing counterpart (97.5 µg/g of dry matter). This may be due to a more efficient extraction in the former due to the presence of oil [21] and due to varietal differences in the tomato batches used for making the two bread types [22]. Broccoli significantly increased the protein content of bread whilst the other vegetables reduced it, and this indicates that vegetables could be used to modulate the protein content of bakery foods. Similarly, the significantly lower total carbohydrate content in the vegetable breads indicate that vegetables could be used to reduce the glycemic load of bread.

Similar to their oil-containing counterparts, the vegetable breads in this study also showed an improved antioxidant profile. The FRAP antioxidant potential showed similar magnitudes as well as trends for oil-free and oil-containing breads, where beetroot and carrot breads showed the highest and lowest antioxidant capacities, respectively. These trends agree with previous reports [23]. However, the HORAC results for the two bread types were notably different where all the oil-containing breads showed higher HORAC antioxidant capacities (50–60 µmol of Gallic Acid Eq/g of bread) compared to the oil-free variants (2–30 µmol of Gallic Acid Eq/g of bread) and this suggests that oil increases metal chelation properties. Although chelators are typically water soluble, some exhibit fat solubility, and the presence of oil may, therefore, improve their activity [24]. Indeed, plant foods have been shown to contain lipid-soluble metal chelators such as citric and ascorbic acid [24,25], and these may have been instrumental in improving the antioxidant capacity of the oil-containing breads compared to their oil-free counterparts. The similar values seen for the oil-containing vegetable breads and their corresponding plain bread (control) suggests that oil has a greater impact on HORAC antioxidant capacity than vegetables and that the former may have been a confounder in this respect. Therefore, the oil-free breads may be better demonstrators of the effect of vegetables on the HORAC antioxidant properties of bread. Similar to the oil-containing breads, broccoli had the greatest effect on the HORAC antioxidant potential in the oil-free breads and this may be due to the activity of glucosinolates [26]. The other vegetable breads also showed significantly greater HORAC antioxidant capacities compared to the control which suggests that their addition improves the metal-chelation properties of bread. The overall data for the oil-free and oil-containing breads suggest that the latter variant is the better model for vegetable breads from an antioxidant potential perspective. 

As expected, the TBARS content was markedly lower in the oil-free breads compared to the oil-containing breads (7–15 vs. 13–103 nmol/g of bread, respectively). Although tomato and broccoli breads showed statistically significant higher levels compared to the plain bread, the overall low concentrations (12–15 nmol/g) suggest that they may not be in amounts that are of practical relevance or concern (compared to the higher levels seen in the oil-containing breads). Beetroot appeared to significantly reduce TBARS levels compared to the control, and this further reinforces its previously demonstrated superior antioxidant effects [3,5]. The present study further showed that vegetables significantly reduced protein oxidation in bread (measured as PCs). The PC levels in the fresh oil-free carrot, beetroot, and broccoli breads were similar to levels in the oil-containing breads [2] (35–66 vs. 44–75 nmol/mg of protein) which suggests that the vegetables reduced protein oxidation independently of the effects of oil. Tomato also attenuated PCs in the oil-free bread (59 nmol/mg of protein) unlike in the oil-containing breads where it appeared to provoke them (73 nmol/mg of protein). Although this confirms tomato’s potential for reducing protein oxidation [27], the divergent results observed for the two bread types warrants further research. TBARS such as Malondialdehyde (MDA) generated from lipid peroxidation have been shown to generate secondary products such as PCs [28], and the elevated TBARS levels in the oil-containing tomato bread may have led to the production of excess PCs compared to in its oil-free counterpart. 

All three textural attributes (hardness, cohesiveness, and gumminess) for the oil-free breads showed lower values compared to the oil-containing breads and this confirms previous reports of lipids improving the softness and crumb characteristics of bread [29]. These inferior values were despite the oil-free breads having a greater moisture content, which indicates a greater role of oil in the textural quality of bread than water. The force required to compress/deform the bread with the molar teeth during chewing is considerably lower for the oil-containing breads after four days of storage (Figure 5) which further shows the positive effects of oil addition in maintaining the long-term softness of bread. Thus, the incorporation of lipids into vegetable breads improves its textural and shelf-quality properties. If oil is to be omitted then the addition of alternative ingredients (e.g., hydrocolloids or gums) with fat-mimetic properties could be considered for improving texture, sensory characteristics, and shelf-life of fat-free baked products [30]. Although fibre from different sources has been documented to improve the structure of bread [31], this effect was not evident in the present study. Similar to its oil-containing counterpart, the oil-free broccoli bread had the poorest loaf volume (Figure 1) which confirms the adverse effects of uncooked broccoli on leavening, possibly due to the negative effects of sulphur compounds (contained in brassicas) on yeast activity [32]. 

Similar to the oil-containing breads, adding vegetables to oil-free breads had no positive impact on macronutrient oxidation during gastro-intestinal digestion. However, as could be expected (in the absence of oil) the overall TBARS levels generated during digestion of the oil-free breads was significantly lower than what was seen in the oil-containing breads (6–470 nmol/g of sample compared to 9–1600 nmol/g of sample, respectively). Similar to what was observed with the oil-containing bread, adding broccoli to oil-free bread significantly provoked TBARS formation during all three phases of digestion. It is clear from this repeatedly observed phenomenon that broccoli induces lipid peroxidation during digestion and this warrants investigation. Future studies should also investigate if this is an occurrence peculiar to broccoli or common to Brassicas. Similar to the oil-containing breads, PCs in oil-free vegetable breads were comparable to the control which suggests that the vegetables did not affect protein peroxidation during gastro-intestinal digestion. Furthermore, the PC contents in the oil-containing and oil-free breads showed similar ranges (70–700 nmol/g of protein). These results collectively indicate that vegetables may be playing a greater role in lipid oxidation during digestion. To our knowledge these two studies represent the first instance that assessed the antioxidant properties of vegetables during gastro-intestinal digestion and more work in this important area is required. 

As highlighted in the first part of this article [2], the absence of a sensory evaluation of the breads is a study limitation. As these studies were primarily carried out as proof-of-concept to determine the nutritional and oxidative implications of adding vegetables to bread we felt that a sensory evaluation was not appropriate at the initial stage. As the results observed in this initial study are promising, the breads will now be assessed for their sensory attributes. 

## 5. Conclusions 

In conclusion, similar to the breads made with the addition of oil [2], adding vegetables also improved the overall nutritional and functional attributes of oil-free breads. The oil-free breads demonstrated a lower antioxidant potential compared to their oil-containing counterparts. Similarly, they showed poorer texture attributes than the oil-containing breads. Compared to the oil-containing breads the oil-free breads were associated with lower lipid oxidation levels both in their fresh form and during gastro-intestinal digestion. Adding vegetables reduced protein oxidation in the fresh oil-free breads but had a neutral effect during gastro-intestinal digestion. The impact of vegetables on macronutrient oxidation during digestion appears to be vegetable-specific with broccoli exacerbating it and the other evaluated vegetables having no effect. Of the vegetables evaluated in the present study, beetroot showed the most promising nutritional and physico-chemical benefits when incorporated into bread not containing added oil.

## Figures and Tables

**Table 1:** Compositional information of the breads.

	Plain Bread	Broccoli Bread	Carrot Bread	Tomato Bread	Beetroot Bread
Moisture	41.2 ± 0.1	43.7 ± 0.2	43.2 ± 0.1	43.2 ± 0.3	43.6 ± 0.1
Protein	14.2 ± 0.1	16.2 ± 0.0 *	13.2 ± 0.1 *	13.9 ± 0.1 *	13.6 ± 0.0 *
Total Carbohydrates	75.9 ± 1.6	68.1 ± 1.0 *	65.8 ± 1.5 *	70.2 ± 1.0 *	69.9 ± 0.3 *
Fat	0.6 ± 0.0	0.8 ± 0.0 *	0.6 ± 0.0	0.8 ± 0.0 *	0.6 ± 0.0
Ash	3.1 ± 0.2	3.9 ± 0.1 *	3.6 ± 0.1 *	3.8 ± 0.1 *	3.7 ± 0.1 *
NSP	5.6 ± 0.2	8.2 ± 0.0 *	7.1 ± 0.0 *	7.5 ± 0.4 *	6.7 ± 0.0 *
α- and γ-Tocopherol	39.0 ± 3.3	104.0 ± 1.8 *	50.7 ± 4.9	106.2 ± 11.3 *	36.3 ± 2.0
α-Carotene	-	-	41.0 ± 1.8 *	1.7 ± 0.1	-
β-Carotene	0.2 ± 0.1	11.6 ± 1.0 *	144.0 ± 6.3 *	29.9 ± 1.6 *	0.3 ± 0.1
β-Cryptoxanthin	-	-	0.3 ± 0.1 *	-	-
Lutein/Zeaxanthin	3.4 ± 0.2	34.5 ± 2.9 *	6.6 ± 0.2 *	9.1 ± 0.5 *	4.5 ± 0.6 *
Lycopene	-	-	-	54.6 ± 3.0 *	-

Data presented are mean ± SD. Tocopherols and Carotenoids are in µg/g of dry matter, Proximate values are g per 100 g of dry matter; NSP = Non-Starch Polysaccharides (Rhamnose, Fucose, Arabinose, Xylose, Mannose, Galactose, Uronic acid); For each nutrient/non-nutrient, values with asterisks are significantly different to plain bread (One-way ANOVA, post hoc REGWQ test, *n* = 3; *p* < 0.05); Blank cells indicate no detectable levels.

**Table 2:** Textural properties of the breads both while fresh and during storage.

	Hardness (N)	Cohesiveness	Gumminess (N)
Day 0			
Plain	4.45 ± 1.2 ^a^	0.67 ± 0.1 ^a^	2.79 ± 0.5 ^a^
Carrot	6.56 ± 0.3 ^a^	0.46 ± 0.1 ^a^	3.12 ± 0.2 ^a^
Tomato	5.01 ± 0.8 ^a^	0.57 ± 0.1 ^a^	3.14 ± 0.1 ^a^
Beetroot	8.37 ± 1.6 ^b^	0.67 ± 0.0 ^a^	5.44 ± 1.3 ^ab^
Broccoli	15.72 ± 1.3 ^c^	0.55 ± 0.0 ^a^	6.90 ± 0.9 ^b^
Day 1			
Plain	5.46 ± 1.7 ^a^	0.61 ± 0.0 ^a^	3.81 ± 0.7 ^a^
Carrot	10.43 ± 0.4 ^ab^	0.46 ± 0.1 ^b^	4.53 ± 0.7 ^ab^
Tomato	8.80 ± 1.1 ^a^	0.47 ± 0.0 ^ab^	3.98 ± 0.8 ^ab^
Beetroot	11.28 ± 0.8 ^ab^	0.52 ± 0.0 ^ab^	5.48 ± 0.3 ^ab^
Broccoli	16.34 ± 3.3 ^b^	0.44 ± 0.0 ^b^	6.81 ± 1.1 ^b^
Day 2			
Plain	8.77 ± 0.4 ^a^	0.55 ± 0.1 ^a^	4.42 ± 0.2 ^a^
Carrot	10.87 ± 0.2 ^a^	0.45 ± 0.1 ^a^	4.44 ± 0.6 ^a^
Tomato	8.90 ± 0.3 ^a^	0.45 ± 0.0 ^a^	4.02 ± 0.4 ^a^
Beetroot	13.33 ± 3.8 ^ab^	0.43 ± 0.0 ^a^	5.71 ± 1.9 ^ab^
Broccoli	20.38 ± 2.4 ^b^	0.48 ± 0.0 ^a^	8.36 ± 0.9 ^b^
Day 4			
Plain	10.9 ± 1.3 ^a^	0.51 ± 0.0 ^a^	5.38 ± 0.9 ^a^
Carrot	11.99 ± 0.2 ^a^	0.37 ± 0.1 ^a^	4.41 ± 0.5 ^a^
Tomato	9.59 ± 2.0 ^a^	0.47 ± 0.1 ^a^	4.10 ± 0.6 ^a^
Beetroot	15.06 ± 0.5 ^a^	0.39 ± 0.0 ^a^	5.78 ± 0.4 ^a^
Broccoli	22.00 ± 2.4 ^b^	0.38 ± 0.0 ^a^	8.32 ± 0.4 ^b^

Values are means ± SD (*n* = 4); values with different superscripts within a column for each day are significantly different (One-way ANOVA, post hoc REGWQ test, *p* < 0.05); Cohesiveness is unit-less.
